# The mindful leader: a review of leadership qualities derived from mindfulness meditation

**DOI:** 10.3389/fpsyg.2024.1322507

**Published:** 2024-03-05

**Authors:** June Borge Doornich, Helen Miller Lynch

**Affiliations:** ^1^Business School, Nord University, Bodø, Norway; ^2^Medical Yoga Sweden of California, Bodfish, CA, United States

**Keywords:** mindful leader, leadership, mindfulness, meditation, neuropsychology

## Abstract

Mindfulness has been practiced by global leaders and companies as an efficient way to build effective leadership. Because of its popularity, plus the lack of a comprehensive theoretical framework that explains it in a leadership context, the research literature has called for a coherent account of the qualities that is derived by those leaders that practice mindfulness. Here, we aim to answer that call, by clarifying *what leadership qualities can develop from practicing mindfulness*. We report on a semi-systematic literature review of extant research, covering 19 research articles published between 2000 and 2021, plus other relevant supporting literature from the disciplines of leadership and neuropsychology. Our proposed framework consists of three main qualities of the mindful leader: attention, awareness, and authenticity. We call them the “three pillars of mindful leaders.” We also propose that mindfulness meditation must be integrated into our proposed framework, as we are convinced that leaders who hope to benefit from these qualities must integrate a regular mindfulness meditation practice into their daily leadership life.

## 1 Introduction

Many influential global leaders are now implementing mindfulness as a daily practice. Examples include Bill Gates, Richard Branson, Oprah Winfrey, and Russell Simmons. Meanwhile, some major companies have begun implementing mindfulness meditation training in their leader-development programs. Examples are Google, with its “search inside yourself” program, and General Mills, with its “finding the space to lead” program.

Even the World Economic Forum has acknowledged mindfulness as a method for developing effective leaders. It now offers morning mindfulness meditations for leaders, typically attending in their black suits and dresses, at the Annual Meeting in Davos.

But while companies are popularizing mindfulness as a way to promote mindful leaders, the research community has yet to settle on a coherent scientific account nailing down the qualities leaders acquire from practicing mindfulness (Brendel et al., 2016; Good et al., 2016; King and Badham, 2018; Rupprecht et al., 2019).

Whereas scholars have suggested compelling frameworks about the qualities leaders develop from mindfulness (Brendel et al., 2016; Ehrlich, 2017; King and Badham, 2018; Stedham and Skaar, 2019), their very variety leaves us with conceptual inconsistency and ambiguity as to just what counts as “qualities of the mindful leader” (Brendel et al., 2016; Van Dam et al., 2018; Zhang et al., 2021). That ambiguity might be explained by mindful leaders being a relative novel phenomenon within academic research (Good et al., 2016).

The research community therefore is calling for more studies to help us agree on what qualities leaders supposedly develop from practicing mindfulness (Brendel et al., 2016; Good et al., 2016; King and Badham, 2018; Daniel et al., 2020).

In this paper, we aim to propose a theory that helps to both identify and explain the qualities of the mindful leader. We therefore address the following research question: *What qualities can leaders develop from practicing mindfulness?* We have conducted a literature review of extant publications to reveal the qualities of the mindful leader (Webster and Watson, 2002; Snyder, 2019). Our review resulted in 19 conceptual and empirical scientific studies. Based on these publications, we suggest and outline in this paper three main qualities that a leader can gain from a formal mindfulness practice: attention, awareness, and authenticity.

Furthermore, when we are engaged in mindfulness as a meditation practice, it lets us re-program both our “hardware” (i.e., our brain) and our “software” (i.e., our mind) (Miyake et al., 2000; Herndon, 2008; Malinowski, 2013; Mohapel, 2018). Neuropsychology has made extensive contribution to unveil the benefits of mindfulness meditation on our brain and mind. As neuropsychology is the science of the relation between our brains function and our mind (i.e., cognition, emotions and behavior), we find it intriguing, interesting, and necessary to integrate a neuropsychological explanation for why these three leader qualities develops when practicing mindfulness as a meditation. Neuropsychology will therefore be woven into our discussions about the qualities of the mindful leader, as it gives us a richer backdrop about how mindfulness meditation can rewire our brain and mind, and thereby foster these qualities of the mindful leader. In our review, we therefore limit the phenomenon “mindfulness,” to be understood as a formal practice of mindfulness meditation.

First, though, we’ll offer a short overview of mindfulness from both a Buddhist and a Western perspective. Then, we’ll outline our methodology for theorizing about qualities of the mindful leader. Our findings about the three qualities of the mindful leader are then presented, and followed by a discussion and conclusion of our study.

## 2 Mindfulness

*“I invite you to take a few moments to find a comfortable position on a chair, or in a cross-legged position on a meditation pillow on the floor.*

*Sit with a straight back, your hands resting on your thighs and with both feet on the floor.*

*Have your chin gently pulled in and the top of your head reaching toward the ceiling.*

*Adopt a posture that can best support your intention to be awake and alert, but relaxed.*

*Now, close your eyes, or, if you prefer, adopt a soft gaze, looking down toward the floor in front of you.*

*Your spine is straight, but not stiff.*

*Take the next few moments to notice your body’s sensation, like where you might feel ease, tension, tingling, or vibration…*

*Now bring your awareness to your breath, wherever it feels the most prominent, perhaps at your nose, chest, belly, or somewhere else.*

*Simply maintain this awareness of your breath, breathing in and breathing out.*

*Without any judgment, simply watch your breath, breathing in and breathing out through your nose.*

*There’s no place you have to go now, and nothing else to do; just be in the here and now, noticing your breath.*

*Occasionally your attention may wander. When this happens, simply acknowledge where it went and then gently bring your attention back to your breath…*

*In a moment or two, we will be coming to an end of this meditation practice.*

*If you have closed your eyes, allow them to slowly open, or if you have softened your gaze, gently let yourself take in your surroundings in the room, and bring this awareness to your next few moments.”*

*The three-minute breathing meditation – scripted by the authors, inspired by scripts from* *Woods and Rockman (2021)**, and* *Bob and Goldstein (2010)*.

Mindfulness (Pali: Sati) is a practice that originates from Buddhism. It aims to promote enlightenment—that is, a state of wisdom about existence, about the way reality appears to us, and the true nature of its appearances. Mindfulness seeks to liberate us from life sufferings that prevent us from reaching what’s understood as enlightenment (Vu and Gill, 2018).

The Eastern Buddhist mindfulness practice began to spread to the West in the mid- 1970’s. Centers were established in the United States and United Kingdom, offering mindfulness programs (Woods and Rockman, 2021) that introduced the practice as a way to promote positive psychological mental health and well-being (Purser and Milillo, 2015; Vu and Gill, 2018). Although “enlightenment” is not the expressed goal of mindfulness training in the West, the aim is still to reach deeper insight into, and reflection about, our mind and how our surroundings appear to us, and to adjust those mental processes and behavioral patterns that are associated with “life sufferings.”

In Buddhism, meditation is far from a “woo-woo thing”; it is basically just a form of “mental training” that increases our capacity for enlightenment and fine-tunes our mental processes and behavioral patterns. There are many different types of meditation in Buddhism. In the present paper, we’ll be speaking about the meditation practice that is integrated in widespread mindfulness programs. Popularly called “mindfulness meditation.” It involves paying attention to experiences in the moment, with openness and acceptance, with less reactivity and judgment. This meditation practice is based on the Buddhist meditation practice called Vipassana.

Vipassana meditation uses the breath as an anchor in the practice. It’s practiced while lying on the floor, sitting on a chair, or simply while walking. But, most often, it is practiced while sitting cross-legged on the floor on a meditation pillow, with a posture that allows for being awake and alert, but relaxed, with your attention focused on your breath and the sensations in your body. The “three-minute breathing meditation” that we scripted to introduce this section is typical of Vipassana meditation.

In recent years, though, “mindfulness meditation” has also been augmented with mindful movements, singing tones (known as chanting), and finger movements (known as mudras), though it still uses the breath as an anchor.

John Kabat-Zinn was one of the early influential scholars that adapted Buddhist mindfulness in a Western academic and practice context. He defined mindfulness as “an awareness that emerges through paying attention on purpose to the present moment and non-judgmentally to the unfolding of experience moment by moment” (Kabat-Zinn, 2003, p. 145).

Other influential scholars define mindfulness similarly, though with somewhat different phrasings. For example, Brown and Ryan (2003) define it as a receptive attention to, and awareness of, present events and experiences, while Bishop et al. (2004, p. 323) define it as “a kind of non-elaborative, nonjudgmental, present-centered awareness in which each thought, feeling, or sensation that arises in the attentional field is acknowledged and accepted as it is.” Shapiro and Carlson (2009, p. 556), meanwhile, define mindfulness as “the awareness that arises through intentionally attending in an open, caring, and discerning way.”

Leadership scholars depart from this accepted definition of mindfulness when they implement it in a leader context. Leaders who practice mindfulness apparently enjoy greater “cognitive and emotional resources that ensure resilience in the face of stress, and the flexibility of mind and learning skills to adapt to a fast-changing employment market and longer working lives” (The Mindfulness Initiatives, 2015, p. 6). There seems to be a widespread agreement that mindfulness can indeed develop particular qualities of the mindful leader (Ehrlich, 2017; King and Badham, 2018; Mohapel, 2018; Stedham and Skaar, 2019).

Conceptual papers and empirical research have proposed various qualities developed by leaders who practice mindfulness. Brendel et al. (2016), suggested that mindful leaders’ qualities are creativity, less trait anxiety, and reduced stress. For Ehrlich (2017), meanwhile, mindful leaders’ have the qualities of spirit (knowing where one is going), emotion (staying with one’s feelings, and accessing the information they contain), mind (paying attention to one’s thinking), body (attending to one’s physical self), connecting (focusing on others), and inspiring (having a vision and passion that motivate others to join in).

King and Badham (2018), meanwhile, contend that the enhanced leaders qualities are individual mindfulness (an increased capacity for being aware of, attentive to, and accepting of experience), individual wisdom (greater reflexivity, relationality, and compassion in adopting and pursuing individual and collective purposes), collective mindfulness (greater adaptability, reliability, and resilience in organizational cultures and systems), and collective wisdom (that enhances consciously responsible, collaborative, and sustainable forms of governance).

Stedham and Skaar (2019), meanwhile, suggested that mindfulness promotes a host of leader qualities—attention, reperceiving, cognitive capacity, cognitive reflexivity, self-regulation of emotions, self-regulation of behavior, self-awareness, social awareness–as well as several leadership behavioral characteristics—empathy, humility, authenticity, transparency, standing back, positivity, and resilience.

While leadership scholars like these are proposing interesting insights into the qualities of mindful leaders, their variety leaves us with conceptual inconsistency and ambiguity as to just what counts as “mindful leaders” (Brendel et al., 2016; Van Dam et al., 2018; Zhang et al., 2021).

Scholars therefore lament the lack of a comprehensive, well-accepted theoretical model that explains what is to be known as a mindful leader and what are their qualities, as it makes the phenomenon not only hard to grasp, due to the lack of an agreed-upon and precise conceptualization, but also difficult to apply in research that allows for methods that ensure validity (Donaldson-Feilder et al., 2019; Rupprecht et al., 2019; Daniel et al., 2020). Scholars are therefore calling for more research on the topic in hopes of finally reaching agreement on exactly what constitutes mindful leaders as a novel theory in the leadership discipline (Good et al., 2016; Lange et al., 2018).

In this paper, therefore, we aim to help answer that need. We have compiled and synthesized a vast corpus of current research on mindful leaders in hopes of proposing a convincing theory about the qualities associated with the mindful leader. In the following section, we outline our approach for building such a theory.

## 3 Methodology for building theory

When novel phenomena get implemented in practice, researchers typically seek to build convincing theories about them, both to explain them and to advance our scientific knowledge of them. Theorizing is often sparked by the lack of a coherent theory that explains practices in real life to help ameliorate real contemporary problems (Wacker, 1998; Shepherd and Suddaby, 2017).

A theory is “*a statement of concepts and their interrelationships that shows how and/or why a phenomenon occurs*” (Corley and Gioia, 2011, p. 12). Theory, in short, is an abstraction and simplification of some observable phenomenon. A theory offers to define the phenomenon and its main concepts or variables and their relations with a set of circumstances where the theory is applied (Wacker, 1998).

A strong theory typically develops over time, as the mounting scientific evidence associated with the phenomenon accumulates into an agreed-upon understanding. These emerging scraps of evidence and explanations posited about the phenomenon allow us to synthesize a coherent and convincing theory (Wagner and Berger, 1985). Coherent theories enable a common understanding of the phenomenon, thereby providing a theoretical framework for still further scientific development that will continue to expand our understanding and effective implementation in practice (Wacker, 1998; Webster and Watson, 2002; Weber, 2003).

When theories about a phenomenon are still in their infancy, theory development can be facilitated by comprehensive literature reviews that single out, and analyze, key scientific contributions that advance our knowledge of a phenomenon (Webster and Watson, 2002). A “literature review” is a well-established research methodology for systematically collecting, analyzing, and synthesizing previous research on a given phenomenon in order to build theoretical models explaining contemporary problems (Webster and Watson, 2002; Snyder, 2019).

Our own literature review, reported here, aims to study the extant knowledge and explanations of the qualities of a mindful leader in hopes to make a leap forward in developing a robust, coherent theory about mindful leaders. We have conducted a semi-systematic review, which normally suffices when one is mapping conceptual models, empirical evidence and theoretical explanations in order to get an overview of key concepts that, when synthesized, build a coherent theory (Snyder, 2019). It also suffices when a phenomenon has been conceptualized differently and studied by various groups of researchers within diverse scientific disciplines (Wong et al., 2013; Snyder, 2019).

We have followed peer-reviewed standards for conducting literature reviews—standards that have been accepted and applied in different academic disciplines (Wong et al., 2013; Aguinis et al., 2017; Snyder, 2019).

We were particularly inspired by the method suggested by Aguinis et al. (2017). Accordingly, in the first step of our review, we defined both its *goal and scope*, and decided on the inclusive and exclusive criteria for articles to review. As for our goal, we aimed to review extant literature discussing mindfulness in a leadership context, with the intention to develop a theory on the qualities that can develop from practicing mindfulness. As for scope, we restricted our review to articles focusing on mindfulness in a leadership context, which were published between 2000 and 2021, since mindfulness only started to emerge in the Western science community in the early 2000’s (Baminiwatta and Solangaarachchi, 2021).

We included both conceptual and empirical articles that focused at least in part on leadership qualities promoted by mindfulness, but excluded articles that mainly focused on how mindfulness could benefit the leaders’ mental health and wellness, such as relieving stress, anxiety, and depression.

Next, as for which journals to scour, we started with the management journals listed in the ABS journal ranking (the Chartered Association of Business Schools). We searched for the words “mindful*,” “leader*,” and “management” in the article abstracts to identify articles for first-order selection. Since we got quite a few hits, we chose to broaden that search, sifting through three large databases: ScienceDirect, Emerald, and PubMed.

After reading the abstracts there, we had to decide whether the articles merited a second-order selection, based on our inclusion and exclusion criteria. When we landed on a pile of relevant articles, we sifted through them individually to remove any that did not meet our review’s goal. Our search left us with 19 research articles that met both our inclusion and exclusion criteria.

Then, using the NVivo program, we analyzed them to learn the main trending concepts that they discussed. Specifically, we ran a *word frequency analysis*. Here, we needed to remove some words that showed up in the list but did not refer to a leadership quality, such as “study,” “research,” “results,” “model,” “experience.” We did the same with words that were too alike—for example, keeping the word “leadership” while removing the word “management,” and also keeping the word “organizations” but removing the word “organization.” Table 1 shows our final analysis of the word frequency that provided us with initial ideas about the leadership qualities of mindful leaders discussed in the literature.

**Table 1:** word frequency analysis of the 19 research articles reviewed in our study.

Word	Count	Weighted percentage (%)
Mindfulness	6,111	5.12
Leadership	5,233	4.82
Practice	1,627	1.05
Performance	975	0.73
Behavior	928	0.80
Self	769	0.71
Emotional	742	0.68
Attention	690	0.54
Psychology	682	0.63
Organizations	671	0.55
Intervention	641	0.57
Awareness	515	0.22
Regulation	496	0.40
Relationship	486	0.45
Transformation	486	0.45
Authentic	444	0.35
Perceptions	405	0.25
Communication	389	0.35
Trust	353	0.21
Response	342	0.30
Ethical	296	0.25
Acceptance	285	0.22
Cognitive	273	0.22
Compassion	259	0.23
Social	244	0.22

Following our NVivo analysis, we scrutinized each article included in our review, in light of the framework laid out by Luft and Shields (2003). We analyzed each article with respect to four questions: What concepts and significant forces are investigated? What are the key findings and arguments? What theoretical approach underpins the study? What methodology is applied?

While compiling and synthesizing current research on mindful leaders, we identified three main qualities that is developed by leaders that practice mindfulness: *attention*, *awareness*, and *authenticity*. This allowed us to categorize the extant literature into three main concepts that explain the qualities typically developed when leaders practice mindfulness.

We then went back to NVivo to make a *text-search analysis* of the three main concepts appearing in the 19 articles. We separately searched for those three concepts, in a broad context, compiling individual documents of the concepts that collected all the texts in the literature mentioning the three concepts. This provided us with transparent, and easily accessible, text documents about the literature’s discussions of the qualities found in the mindful leader. In Table 2, we show which of the main qualities that the articles have mainly covered in their discussions.

**Table 2:** The main qualities addressed in the 19 research articles reviewed in our study.

Reference	Year	Journal	Title	Method	Atte.	Awar.	Auth.
Arend et al.	2019	Front. Psychol.	Mindfulness and leadership: communication as a behavioral correlate of leader mindfulness and its effect on follower satisfaction	Quantitative			X
Baron	2016	J. Mana. Psychol.	Authentic leadership and mindfulness development through action learning	Mixed method			X
Brendel et al.	2016	J. Manag.	Cultivating leadership dharma. Measuring the impact of regular mindfulness practice on creativity, resilience, tolerance for ambiguity, anxiety, and stress	Quantitative		X	X
Burmansah et al.	2020	EU-JER	Mindful leadership: the ability of the leader to develop compassion and attention without judgment—a case study of leader of Buddhist higher education institute	Qualitative			X
Chesley and Wylson	2016	J. Chang. Manag.	Ambiguity: the emerging impact of mindfulness for change leaders	Mixed method	X	X	
Decuypere et al.	2018	Front Psychol	When mindfulness interacts with neuroticism to enhance transformational leadership: The role of psychological need satisfaction	Quantitative		X	
Ehrlich	2017	Organ. Dyn.	Mindful leadership: Focusing leaders and organizations	Conceptual	X	X	X
Hunter and Chaskalson	2013	Book chapter	Making the mindful leader: Cultivating skills for facing adaptive challenges	Conceptual	X	X	
Kersemaeker et al.	2020	BMC Med. Educ.	Effectiveness and feasibility of a mindful leadership course for medical specialists: a pilot study	Quantitative			X
King and Badham	2018	Asia Pac. J. Hum. Resour.	Leadership in uncertainty: The mindfulness solution	Conceptual	X		X
King and Harr	2017	Asia Pac. J. Hum. Resour.	Mindfulness and job performance: a study of Australian leaders	Quantitative	X		X
Lange and Rowald	2019	Gr Interakt Org	Mindful leadership: evaluation of a mindfulness-based leader intervention	Quantitative		X	X
Lippincott	2018	Leadersh. Organ. Dev. J.	Deconstructing the relationship between mindfulness and leader effectiveness	Qualitative		X	X
Mohapel	2018	HMF	The neurobiology of focus and distraction: The case for incorporating mindfulness into leadership	Conceptual	X		
Nübold et al.	2020	J. Bus. Psychol.	Be(com)ing real: a multi-source and an intervention study on mindfulness and authentic leadership	Qualitative			X
Reitz et al.	2020	J. Manag. Dev.	Developing leaders through mindfulness practice	Qualitative		X	X
Rupprecht et al.	2019	Front Psychol.	Mindful leader development: How leaders experience the effects of mindfulness training on leader capabilities	Qualitative	X	X	X
Stedham and Skaar	2019	Front Psychol.	Mindfulness, trust, and leader effectiveness: A conceptual framework	Conceptual		X	X
Verdorfer	2016	Mindfulness	Examining Mindfulness and Its Relations to Humility, Motivation to Lead, and Actual Servant Leadership Behaviors	Quantitative	X	X	X

Below, we’ll present the findings of our literature review, and we’ll structure our outline of the review, based on these three qualities marking the mindful leader.

## 4 Literature review

Our review of the 19 scientific conceptual and empirical articles on the qualities of mindful leaders shows that the leaders sharpen and sustain their attention, they enhance their level of awareness, and they bring authenticity into their leadership. When we in the following outline these qualities of the mindful leader, we also include other studies from disciples other than leadership to get greater support on our theoretical framework. In particular, we include studies from neuroscience on mindfulness meditation to explain how these qualities are developed.

### 4.1 Attention

Given the seemingly unlimited information and events calling for our attention, our capacity to attend to all of these competing triggers is naturally limited by our own powers of cognition. We are constantly choosing, consciously or unconsciously, between all the competing attention-triggers, and have to decide where and when to allocate a clear and vivid form of attention.

Our review of the literature shows that mindfulness enhances leaders’ capacity to be *intentionally present* to what is taking place in the moment, and to maintain a *sustained attention* over a significantly longer period of time, despite frequent distractions. In what follows, we will elaborate on these main qualities of attention.

#### 4.1.1 Intentional presence

All any of us truly have is the present moment. When we are present in the moment, we inevitably achieve richer experiences because we are “*fully present to what is taking place*” (Schuyler, 2010, p. 21). But our mind tends to wander between the past and the future, drifting away from experiences in the moment. We replay and ruminate over previous experiences—wondering about what could have been, and what we could have done. We also imagine and plan for future scenarios—wondering about what might happen, and rehearsing how we might then respond. During half of our waking day, our mind typically wanders to places that are not strictly relevant to the situation or relation at hand (Killingsworth and Gilbert, 2010).

We also let our attention get derailed by trivial interruptions in our surroundings. Ehrlich (2017) tells about how leaders at Microsoft regularly find themselves interrupted, indeed some 10–30 times an hour, often by mundane things like emails and instant messages. It would then take these leaders 10–25 min to get back on task, meaning that they were rarely fully intentionally present at the issue at hand.

When leaders mentally wander away like this, they risk missing out on important information and cues about what is occurring. Mind-wandering might lead to inappropriate and incomplete responses, and restrict their relational orientation due to the leaders’ semi-presence. When, on the other hand, leaders are fully present in whatever is taking place, they are better equipped to recognize, interpret, and act more appropriately to what is occurring.

Neuroscience informs us that how we attend to our life situations is governed by two neurological systems in our brain (Weissman et al., 2006). One is the *default mode network* (DMN), it’s activated when we daydream, when our mind wanders, when we attend passively to what is occurring, and when we turn our mind inward to ourselves. The other, and higher, system is the *executive control network* (ECF), which needs to suppress the default DMN to be activated, so as to evoke an active, purposeful presence to what is occurring, as when we engage in cognitive-demanding reflections and regulations, and when we deliberately focus our mind on our surroundings.

Mindfulness is shown to help activate the ECF of the brain, thereby improving leaders’ capacity to be intentionally present in what we call the “*here and now*” (Herndon, 2008: 32; Mohapel, 2018). Our brain’s DMN, which tends to mindlessly wander away from the present, is therefore significantly reduced when practicing mindfulness (Hunter and Chaskalson, 2013). Even so, our mind will still be inclined to wander, since the brain’s default mode keeps running to other places than the present. But mindful leaders are better equipped to control and regulate where their mind places its attention.

While neuropsychology convincingly demonstrates that mindfulness meditation practice helps us develop intentional presence to what is occurring, studies find significant variations in how much practice time is required. Baer et al. (2012), for example, found that it typically takes 6 weeks of regular practice. Gao et al. (2018), meanwhile, less optimistic, found that it takes 3 months. Lange and Rowald (2019), for their part, found that these capacities can be developed after a seven-hour mindfulness intervention, followed by 5 minutes of mindfulness meditation practice each working day for 2–3 months.

Mindfulness meditation can also teach leaders to let thoughts and emotions arrive without reacting to them, getting carried away by them, or wanting to change them (Vago and Silbersweig, 2012). This is achieved by better acknowledging that our emotions and “*thoughts are seen as passing events in the mind rather than as inherent aspects of self or as necessarily valid reflections of reality*” (Teasdale et al., 2002, p. 285). This response, or nonresponse, is called “non-reactivity.” It’s a central concept within both Buddhist and Western mindfulness; it explains our ability to allow our thoughts and emotions to arrive without any immediate reactions to them and without getting absorbed by them (Bishop et al., 2004; Shapiro et al., 2006; Hölzel et al., 2011), and to allow external experiences to occur without our hastening to evaluate, control, and react to them (King and Harr, 2017).

Thus, mindful leaders are able to detach themselves from their immediate interpretations and meaning-making of issues at hand (Kabat-Zinn, 2003; Rupprecht et al., 2019). Their mindfulness training reduces their DMN’s automatic tendency to turn their mind inward, which would otherwise cause them to identify too closely to what is occurring. Instead by activating their ECF, they engage in everything with less ego involvement and ego defensiveness, both of which otherwise can quickly distort their experiences with subjective biases (Verdorfer, 2016; King and Badham, 2018).

Significantly, these leaders are able to call upon a broader scope of potential responses, and then consciously choose how they react, thus making more informed choices (Hunter and Chaskalson, 2013; Brendel et al., 2016; Chesley and Wylson, 2016; King and Harr, 2017). This comes in handy in virtually any leadership situations, as they are better equipped to mindfully evaluate the appropriate response before committing to it.

When we are intentionally present in the moment, we further develop an attitude of not judging our experiences. Non-judgment, like nonreactivity, is also a central concept within the Buddhist and Western mindfulness literature. Non-judgment allows for an *open and curious* approach to whatever leadership situations and relations we encounter (Stedham and Skaar, 2019). Although we will still experience judgments, mindfulness makes us better at noticing when our judgmental mind runs our thoughts (Brown and Ryan, 2003; Kabat-Zinn, 2003), since mindfulness meditation activates our mind’s engagement in cognitive reflection and regulations (Hunter and Chaskalson, 2013; Mohapel, 2018).

Mindfulness ensures that leaders are more willing to allow for new understandings of situations and relations (Hunter and Chaskalson, 2013), and to notice relevant information and cues that are available, and also to look for alternative ways of understanding their leadership experiences (Chesley and Wylson, 2016).

Mindful leaders are less constrained by preconceived beliefs and biases, which inhibit growth and learning, and which can keep them from grasping how circumstances actually appear (Kabat-Zinn, 2003; Shapiro et al., 2006; Hunter and Chaskalson, 2013). They are also more aware of how quick and reflexive judgments, aptly called “snap judgments,” can lead to inaccurate and incomplete interpretations of experiences (Dane, 2011; Hafenbrack et al., 2014).

Neuroscience demonstrates that mindfulness meditation can help reduce the DMN’s tendency to automatically engage in reactive and judgmental thoughts and behavior; it does so by turning our mind away from internal ego-centered events, and by then evoking an intentional presence with curiosity toward one’s surroundings. Studies show that when we engage in various breathing techniques based on Vipassana meditation, such as abdominal breathing, nostril breathing (single, alternate, or double), or forceful or vocalized breathing, we gently bring our mind to the issue at hand, and reduce reactive and judgmental responses, at any time, immediately (Zaccaro et al., 2018).

When we practice abdominal breathing, with anything from a 10-min session of deep breathing—being 4–10 breaths per minute, compared to the normal range of 10–20 breaths per minute—to just a short, 2-min session of deep breathing (Zaccaro et al., 2018), we bring our mind back to what is occurring in the moment, with the presence of curiosity, with less reactive and judgmental thoughts and behavior.

In summary, the literature informs us that mindfulness practice enhances leaders’ capacity for being intentionally present to whatever is occurring in the moment, and with the healthier attitudes of non-reactivity and non-judgment.

#### 4.1.2 Sustained attention

“Sustained attention” denotes the ability to focus on specific stimuli over a longer period of time. More precisely, it is “*the ability to self-sustain mindful, conscious processing of stimuli whose repetitive non-arousing properties would otherwise lead to habituation and distraction*” (Robertson et al., 1997, 747). Recall that we all face seemingly unlimited triggers competing for our attention. But our cognitive capacity restricts us from attending to all or even most of those triggers. Mindfulness, however, builds our capacity for sustained attention when necessary, ignoring information and events that have nothing to do with the issue at hand, but still recognizing when triggers ought to be brought into attention.

While neuropsychology informs us that mindfulness meditation practice enhances our sustained attention (MacLean et al., 2010; Malinowski, 2013), studies again find variations in how much practice time is required. MacLean et al. (2010), for example, studied participants in a 3-month-long retreat at the Shambhala Mountain Center who practiced Shamatha meditation (akin to vipassana meditation) for 5 hours a day, and found that their sustained attention was strengthened. Moore et al. (2012), meanwhile, found that a 3-h introduction to mindfulness meditation, followed by 10 min of breathing meditation at least 5 days every week for 16 weeks improved participants’ sustained attention.

Even given such variations in the frequency of mindfulness meditation, leaders who practice it can expect to enhance their ability to self-sustain mindful focus on the tasks at hand (Robertson et al., 1997). Mindfulness meditation is most often practiced longer than the “three-minute breathing meditation.” Indeed, the practice can last 20–50 min. One example of a longer meditation technique is the “body scan meditation.” It’s performed while lying on a floor on a soft yoga mat, using the breath to direct one’s focus on various body parts, and noticing whatever thoughts and sensations then arise, but still maintaining focused attention on the breath and the body to sustain attention on what is occurring internally (Bob and Goldstein, 2010; Woods and Rockman, 2021).

Practicing sustained attention, such as the “body scan meditation,” teaches leaders to maintain their attention on the issues at hand, and to create for themselves undisturbed moments of concentration, with a deep focus on what is to be accomplished, by “tuning out” triggering events that aren’t relevant to accomplishing the given tasks. They build the capacity to deliberately sustain attention by maintaining vigilance over time, despite interruptions and changing conditions (Barel and Tzischinsky, 2020).

Meanwhile, mindful leaders also develop the capacity to be simultaneously alert to triggers that stem from their own thoughts, or from their surroundings, even while in deep focus. Neuropsychology shows that mindfulness meditation improves our ability to notice and evaluate triggering information and events even while upholding a sustained attention, and to choose when, and when not, to react to the competing triggers (Miyake et al., 2000; Malinowski, 2013). The improved function of the ECN also enhances our capacity to switch between multiple tasks—particularly, to switch between a shallow attention on the frequent fragmentation of triggers, and a deep, sustained attention on our primary tasks at hand (Miyake et al., 2000).

Mindfulness, therefore, improves leaders’ capacity to switch their attention spontaneously to triggers that require their immediate, if brief, attention, before returning to a deep focus on the primary issue (Farb et al., 2007; Rupprecht et al., 2019). In today’s work environment, leaders constantly need to shift their attention and respond to short and often random events that requires a response (Ehrlich, 2017). That requires a heavy energy consumption by our ECN neurons as we need to “light their fire.” When these neurons are repeatedly exposed to significant levels of stress, “their fire can burn out.” Prolonged stress over time, and even a mild, brief stress situation, can cause a rapid and dramatic loss in the function of these ECN neurons (Arnsten, 2009).

When any of us feel overwhelmed by such stress, it leads to a “flight or fight” response that activates our *sympathetic nervous system* (SNS); this in turn causes further ripple-effects in our body—with increased secretions of our adrenaline glands (adrenaline and noradrenaline) that release catecholamine, leading to high blood pressure, disrupting our homeostatic mechanisms by increasing our heart rate, tightening our muscles, and making our breath quicker and more shallow, all of which finally reduce our function of the ECN, hence cripple our capacity to pay attention (Soares et al., 2013).

Neuroscience shows that mindfulness meditation reduces our stress response (Hölzel et al., 2011) by strengthening the activity in our *parasympathetic nervous system* (PNS), which guides our response to “rest and digest” when triggering events bombard us. That reduces our adrenaline glands’ activity, which will lower our blood pressure, slow our heart rate, relax our muscles, and make our breath both slow and deep, all of which enhance our attention surplus (Soares et al., 2013). Hölzel et al. (2011), for example, found that participants in an 8-week MBSR program improved the neural activity in their PNS, thereby reducing their stress level; these researchers explained that the “body scan meditation” in particular reduced the participants’ stress. Wasylkiw et al. (2015), meanwhile, found that participants attending a 1-week mindfulness-based intervention reduced their stress level, and were also able to sustain that low 8 weeks later.

The research informs us that mindfulness practice enhances leaders’ capacity for sustaining their attention over time, for being alert to spontaneous triggering events that require immediate response, and for making good choices as to which triggers to respond to, while also being able to return to those tasks that require a deep focus with sustain attention over a longer period of time.

### 4.2 Awareness

“Awareness” denotes heightened insight and clarity, making for a broader overview and deeper understanding of the complexities, contradictions, and challenges of whatever is taking place. Awareness provides us insight into our own thoughts and feelings, behaviors and emotions, and toward the multiple dimensions of our surroundings. Awareness is our capacity to present to what is taking place in the moment.

Our literature review found that mindfulness builds leaders’ capacity to engage in slower, reflective, *conscious mind* processes about their leadership, while being aware of their tendency to engage in faster, automatic subconscious processes. It also builds their capacity to engage in *metacognitive* processes that let them revise their thinking and behaving in leadership. In the following, we will discuss these two main qualities of awareness.

#### 4.2.1 Conscious mind

Neuroscience explains that our level of awareness occurs at one of two levels, namely, our conscious and subconscious mind. Our subconscious mind operates fast, mainly from instinct and intuition, with a low awareness and effortless cognitive processing of experiences. Subconscious responses to experiences are automatic, habitual, and associative, dispensing with any further reflections and interpretations of the appropriate way of thinking and behaving; all of that is governed by regions in our DMN. Our conscious mind, meanwhile, operates much more slowly, making critical analyzes and rational and logical interferences from our experiences, all of which make higher demands on our cognitive processes. A conscious response to experiences is reflexive, controlled, and dissociative, with deep reflections and interpretations requiring that we activate regions in our brain’s ECN.

Our subconscious mind will have built a web of beliefs about how best to lead in any given situation. How we perform our role and responsibility will typically draw on unquestioned beliefs that are stored in memory and retrieved when we encounter familiar and associated experiences. Our programmed beliefs shape how we act and behave in both small and big leadership experiences. In sum, they make our leadership instinctive, automatic, and habitual.

But the subconscious mind, being ever so efficient, might be effective during times of stability, it will not necessarily function well in times of change and transitions. Certainly, leaders often encounter novel problems that cannot be solved with already established beliefs (Hunter and Chaskalson, 2013). Leaders who mindlessly interpret and react to novel problems from automatic and habitual patterns can apply maladaptive solutions when these patterns no longer fit the novel conditions of the current problem (Baer et al., 2006; Hunter and Chaskalson, 2013). Relying on automatic and habitual patterns also reduces leaders’ capacity for innovation, as it reduces reflexivity and critical interpretations and approaches to experiences that diminish their perception of possibilities and responses. In such cases, like everyone else, they are limited by their settled beliefs.

The research literature shows that mindful leaders can significantly improve their capacity for awareness—that is, their insight and understanding about themself, including their habitual and automatic patterns of thinking and behaving (Moore and Malinowski, 2009; Lippincott, 2018; Mohapel, 2018).

Neuroscience informs us that when we are reflecting on ourselves in present-moment experiences, including self-evaluation, self-regulation, and self-knowledge—that is, engaged in *experiential self-awareness*—we activate regions in the ECN of our brain (Schmitz et al., 2004). When, on the other hand, we reflect upon past experiences, of episodic memories from the personal past, that stand as important in our life, and when we construct an autobiographical self, based on the past and the imagined future—that is, engaged in *narrative self-awareness*—we activate regions in the DMN of our brain (Newen and Vogeley, 2003; Lieberman and Eisenberger, 2005; Tacikowski et al., 2017).

Studies within neuropsychology, by among others, Farb et al. (2007), found that participants attending an 8-week MBSR program strengthened their experiential self-awareness, with improved capacity to monitor, and to create, knowledge about the self during momentary experiences. It also strengthened their narrative self-awareness, with greater insight into their automatic and habitual sense of self, including their trait adjectives—that is, their consistent personal characteristics that span across time and space. The program also improved participants’ recognition of when they engaged in experiential versus narrative self-awareness. Lutz et al. (2016), meanwhile, found that experienced meditators, with at least 3 years’ experience, and with a minimum of 1 year’s practice of Vipassana mediation, who practice a minimum of 1 h per week, enhanced their experiential self-awareness, while at the same time reduced their tendency for abrogating narrative self-awareness.

The conceptual papers by Schuman-Olivier et al. (2020) and by Vago and Silbersweig (2012), for their part, particularly emphasize that mindfulness meditation promotes self-awareness—specifically, greater insight into our web of beliefs, and also into our automatic and habitual subconscious thoughts and behavior, which further helps to reduce our often distorted and biased beliefs.

Mindful leaders engage in reflections and observations about their ways of thinking and behaving in leadership (Hunter and Chaskalson, 2013; Seitz and Angel, 2020), aiming to gain insight into the origin of their subconscious web of beliefs that fashion how they perform leadership in any issue at hand (Bishop et al., 2004).

Mindful leaders are both curious and careful about how they perceive and interpret experiences, and challenge themselves to explore alternative and novel points-of-view that reduce their automatic, habitual beliefs and biases in their leadership, all of which bring them closer to the actual realty of their experiences (Bishop et al., 2004; Lange and Rowald, 2019). These leaders are able to draw on a greater spectrum of possibilities for how things appear to them; that frees them to let go of any settled expectations of how things should be and to put aside their automatic, habitual subconscious leadership (Hunter and Chaskalson, 2013; Decuypere et al., 2018; Lange and Rowald, 2019).

In sum, mindful leaders are capable of noticing their subconscious leadership style—namely, their automatic, habitual way of thinking and behaving during momentary experiences—by being conscious of how their web of beliefs and biases, which they have narrated over time based on the past and imagined future, might restrict their performing more efficiently in their everyday leadership experiences.

#### 4.2.2 Metacognition

Metacognition is a higher order of thinking. It’s a cognitive process where we lift our thoughts and feelings, behavior and emotions, to a meta, or higher order, of reflection that goes beyond our immediate observations of the self. While “cognition” refers to the mental processes of perceptions, interpretations, and reasoning, “meta” means “beyond” or “behind,” referring to our way of “*thinking about thinking*” (Flavell, 1979, p. 906).

Metacognition involves, then, our knowing about own cognitive processes and our capacity to regulate and change our basic mental models and beliefs (Flavell, 1979). Metacognition is a supremely self-conscious, self-critical way of learning and transforming, involving our ability to know how to analyze, to draw conclusions, to learn from, and to put into practice what we have learned. Metacognition, then, lets our conscious mind overrun our subconscious automatic and habitual way of thinking and behaving.

Mindfulness meditation gives leaders insight into themself, with the ability to change their web of beliefs and biases about leadership, and to gain broader, more accurate, and more sophisticated world views (Hunter and Chaskalson, 2013; Good et al., 2016). Mindful leaders are therefore better equipped to view their own beliefs as just that—as subjective, constructed notions—rather than as beliefs reflecting some objective reality or truth (Ehrlich, 2017).

Metacognition opens leaders’ awareness to multiple points-of-views and possible interpretations of experiences (Dane, 2011; Hafenbrack et al., 2014), to include, and process, more relevant, accurate, and objective information for interpretation (Herndon, 2008; Karelaia and Reb, 2015). Leaders thus become better at drawing inferences from broader available data about what is occurring, thereby limiting the loss of potentially relevant data and the likelihood of misinterpretations, without being misled by their biased mental models and beliefs (Herndon, 2008; Hunter and Chaskalson, 2013). From broader scanning of information, we gain greater context-relevant interpretation of experiences that result in appropriate adaptive responses to what is actually occurring in the moment (Baron, 2016; Rupprecht et al., 2019; Reitz et al., 2020) This helps ensure that leaders make fewer perceptual failures, since they now realize that “*the distinction between actual events and the interpretation of events is not always obvious”* (Chaskalson, 2011, p. 95).

Mindfulness meditation teaches leaders to “*take a few moments.*” That entails a mental pause allowing for a breathing space between interpretations and responses to our leadership experiences. This mental pause improves our capacity to reflect upon ourselves during momentary experiences—that is to say, experiential self-awareness (Schmitz et al., 2004). It also helps to draw inferences between the momentary reflections of experiences, and the leadership stories we tend to build on our web of beliefs and biases—that is to say, narrative self-awareness (Newen and Vogeley, 2003; Lieberman and Eisenberger, 2005; Tacikowski et al., 2017).

The metacognition developed from mindfulness meditation allows for a space between stimuli and response, which in turn allows for a better assessment of experiences with cognitive flexibility—i.e., *“the human ability to adapt cognitive processing strategies to face new and unexpected conditions”* (Moore and Malinowski, 2009, 177)—allowing leaders to choose interpretations and responses from a broader repertoire (Moore and Malinowski, 2009; Brendel et al., 2016; Decuypere et al., 2018), and to develop new, more sophisticated beliefs about leadership (Shapiro et al., 2006; Jankowski and Holas, 2014; Verdorfer, 2016; Stedham and Skaar, 2019).

Mindfulness meditation equips leaders with the capacity for receptiveness and openness to spontaneous, ongoing, and changing circumstances in their surrounding environment (Bishop et al., 2004; Brown et al., 2007). They are curious to explore different points-of-view and interpretations about what is occurring, thanks to their greater acceptance of ambiguity and complexities, and enjoy the meta-cognitive capacity to change their own sedimented beliefs about issues at hand (Chesley and Wylson, 2016).

In sum, the literature informs us that mindful leaders reflect and interpret at a meta level, giving them greater flexibility in how they both think and behave as leaders, and to make necessary and sustainable changes in how they perform their leadership.

### 4.3 Authenticity

Authenticity denotes our integrity, which involves acting true to our true core beliefs and values. Authentic persons have learned to accept themselves, and thus they enjoy the comfort and courage to act from their true-self in all their varied experiences (Avolio and Gardner, 2005). Authenticity is a deeply embedded construct that unfolds itself over time, imparting a feeling of freedom, autonomy, and spontaneity. Authenticity in leadership is formed by the many situations and relations we have encountered in life and leadership, especially by those key crucible events that have made a deep impact on who we are and how we believe we ought to perform as a leader (Avolio and Gardner, 2005).

Our review shows that mindfulness enhances leaders’ insight into their own core *beliefs and values*, and strengthens their integrity in leadership. It also promotes greater *empathy and compassion* toward both ourselves and our organizational relations.

#### 4.3.1 Beliefs and values

Our beliefs represent our deepest assumptions about ourselves, others, and the wider world that we are a part of. Beliefs are our enduring and unquestioned views of reality and our primary convictions about our thoughts and feelings, as well as our perceptions and interpretations about our experiences (Connors and Halligan, 2015). They are regarded as subjective constructs that create a coherent and consistent representation of how we perform our leadership.

Values, meanwhile, refers to what we prefer and regard as meaningful in our life. They motivate and justify our attitudinal and behavioral standpoints and decisions (Rohan, 2000; Schwartz, 2012). Our values transcend the various situations and relations we encounter, creating a sense of continuity and stability in otherwise complex and chaotic leadership experiences. They become our internal compass that guides our actions and behaviors, having a significant impact on how we lead and navigate through leadership experiences.

Our beliefs and values are sedimented into our subconscious mind (Bargh et al., 2001; Seitz and Angel, 2020)—that is, narrated as a meaningful part of our autobiographical self, based on our past experiences and expected future scenarios (Newen and Vogeley, 2003; Lieberman and Eisenberger, 2005; Tacikowski et al., 2017). They govern our responses to internal and external experiences, and often appear in our neural and physiological reactions to experiences (Namkung et al., 2017; Chen et al., 2021).

Neuropsychology shows that mindfulness strengthens our interoception of our authentic self, including our sedimented beliefs and values—interoception being our brain’s ability to catch and process neural and physiological signals (Farb et al., 2007; Donaldson-Feilder et al., 2019; Schuman-Olivier et al., 2020). Interoceptive signals flow to our brain from a host of diverse neural pathways, such as the sympathetic nervous system, the vagus nerve, and the insular cortex, all of which allow us to sense, interpret, and regulate signals from within ourselves (Craig, 2002; Chen et al., 2021). Mindfulness meditation is shown to awaken our interoception to our neural and physiological states of the body, thus giving us greater insight into the self, in response to internal and external experiences (Farb et al., 2007; Schuman-Olivier et al., 2020).

The “*three-minute breathing meditation,*” for instance, often guides us to notice our own pulse; when resting quietly but awake on a meditation pillow, we can feel the sensation of our pulse creating movement in our body—narrowing our sensations of the pulse in our chest, and then to broaden our sensation of the pulse in our whole body (Bob and Goldstein, 2010; Woods and Rockman, 2021). This practice teaches us to notice interoceptive signals in our body that flow to our brain, informing us about our bodily state in our daily experiences (Farb et al., 2007; Schuman-Olivier et al., 2020).

When leaders gain greater interoception to neural and physiological reactions to experiences, they are better equipped to notice their immediate responses, and to then control, regulate, and refine their actionable intentional responses to their leadership experiences (Karssiens et al., 2014). Mindfulness meditation teaches leaders to regulate neural and physiological reactions to experiences—for instance, when confronting a situation that triggers a fight-or-fight response, the introspective insight allows the leader to control their immediate bodily responses, perhaps by using the breath to lower their heartbeat, thereby withdrawing the bloodstream from their muscles, which activates their parasympathetic nervous system for a more rest-and-digest response to the experience (Hölzel et al., 2011; Soares et al., 2013).

Mindfulness is found to promote greater authenticity in leaders (Baron, 2016; Zhang et al., 2021), and to achieve particular insight into their embedded beliefs and values, thanks to their introspection (Brown and Ryan, 2003; Baron, 2016; Verdorfer, 2016; Stedham and Skaar, 2019; Nübold et al., 2020). These leaders translate their beliefs and values into how they perform their leadership—how, for example, they make judgments, evaluations, and decisions (Rokeach, 1973); how they respond to situations and relations at hand (Rohan, 2000).

Mindful leaders prove more attentive and accepting, with the courage to act in congruence with their true self (Brown and Ryan, 2003; Nübold et al., 2020). With such a high integrity to own convictions, mindful leaders ensure a greater predictability in how they perform their role and leadership priorities, with transparent intentions (Baron, 2016; Stedham and Skaar, 2019). While, over time, we all grow, hopefully improve, and change as individuals, we nonetheless have an authentic self that remains at least slightly consistent all the while (Walumbwa et al., 2008; King and Harr, 2017).

Although mindful leaders demonstrate high integrity toward their embedded beliefs and values, they still have the cognitive flexibility to change their convictions (Vago and Silbersweig, 2012). Mindfulness makes leaders question their own embedded assumptions and convictions, reducing the trap of being misled by those embedded beliefs and ways of perceiving and interpreting leadership experiences (Walumbwa et al., 2008; Vago and Silbersweig, 2012). Mindfulness can help reduce our often distorted, dysfunctional beliefs about what we experience, and help us recognize our own creative fantasy (Vago and Silbersweig, 2012). Mindfulness reduces our continuing reinforcement of our automatic cognitive schemas, making possible a more accurate interpretation of ongoing experiences.

In sum, the literature informs us that mindful leaders are authentic, which is to say always true to their own beliefs and values, yet with sufficient integrity and courage to be willing to challenge their convictions.

#### 4.3.2 Empathy and compassion

Empathy in leadership denotes a leader’s sensitivity to others’ feelings and emotional states, and the ability to fashion an appropriate response to those same feelings. Compassion, meanwhile, is empathy combined with the desire to help and act on others’ behalf, so as to relieve their struggles and suffering. Both psychological concepts are rooted in the leader’s desire to mindfully relate to, and understand, others’ perspectives and experiences.

Neuropsychology informs us that empathic and compassionate behavior toward ourselves and others is linked to the activity in the insular cortex in our brain. It is triggered by interoceptive signals from our bodily state, raising emotional awareness in our mind that enhances our empathic and compassionate responses to the situation or relation at hand (Craig, 2002; Namkung et al., 2017). Mindfulness is found to strengthen our activity in the insular cortex, which strengthens our emotional awareness and leaves us with improved empathy and compassion for ourselves and others. Studies by Farb et al. (2007), plus Shapiro et al. (1998), showed that participants at an 8-week MBSR program increased their activation in the insular cortex, resulting in a significantly improved capacity for empathy and compassion.

The “*three-minute breathing meditation*” often guides us to focus on the sensations from our surroundings, and then to focus on the sensations of our body, our breath, and our mind, before extending our attention to the surroundings again, and finishing the practice by *“taking in the surroundings of the room, bringing this awareness to your next few moments.”* (Bob and Goldstein, 2010; Woods and Rockman, 2021). This movement of our attention, between ourselves and our surroundings, teaches us to be observant and present in both our relationships and our further surroundings. Our introspection to the authentic self is not occurring in a capsule apart from our social world; it occurs in relation to the social world that we are a part of. Mindfulness meditation reduces our egocentric tendencies and opens our attention and awareness toward others, helping to create transparent relationships (Good et al., 2016; Verdorfer, 2016; Rupprecht et al., 2019; Kersemaeker et al., 2020; Reitz et al., 2020).

Mindfulness fosters a genuine and pure empathy for ourselves and others in our leadership, all of which build transparent, quality relationships in the organization (Good et al., 2016; Lippincott, 2018; Lange and Rowald, 2019; Zhang et al., 2021). Mindful leaders are empathic and compassionate toward organizational relations by being open and aware of others’ states of being, thinking, and feelings (Shapiro et al., 1998; Rupprecht et al., 2019; Reitz et al., 2020). They are mindful observers and listeners, without judgment and reactivity, and calming the need to react and express their own perceptions and interpretations of issues at hand (Arendt et al., 2019; Rupprecht et al., 2019).

Mindful leaders’ attention and awareness toward others allows for a more accurate and unbiased understanding of their points-of-views, and their embedded beliefs and values (Verdorfer, 2016; Arendt et al., 2019; Rupprecht et al., 2019; Stedham and Skaar, 2019; Nübold et al., 2020; Zhang et al., 2021). These leaders are open toward conflicting perceptions and interpretations of issues at hand due to fundamental variations in others’ understanding of reality, and appreciate differences in order to make richer and more accurate decisions and actions in leadership (Wasylkiw et al., 2015).

Mindful leaders are also compassionate toward organizational relations (Bishop et al., 2004; King and Badham, 2018). They are able to identify with, and serve, others when needed on their own terms, and to adjust their own behavior to the needs of others (King and Badham, 2018; Rupprecht et al., 2019; Burmansah et al., 2020; Nübold et al., 2020). They show a readiness to respond when they sense that others are struggling, and they seek, without egocentric intentions but with genuine interest, to help heal the struggles and suffering of others, and to allay their fear of rejection (King and Badham, 2018; Burmansah et al., 2020). Mindful leaders have a greater interpersonal attunement to understanding others’ internal state, thoughts, and feelings (Stedham and Skaar, 2019), making them better at guiding others through difficult periods (Ehrlich, 2017; Rupprecht et al., 2019).

In summary, the literature informs us that mindful leaders show empathy and compassion for themselves and others, building transparent and trust-based relationships.

## 5 Theoretical framework: the three pillar of the mindful leader

In our literature review, we have sought to offer a theory about the qualities of the mindful leader. Our proposed theoretical framework consists of three main concepts that explain the qualities of mindful leaders: attention, awareness, and authenticity. Inspired by those concepts, we coined our suggested theoretical framework on mindful leaders as “the three pillars of the mindful leader”—a metaphor explaining the three main concepts recurring in the extant research literature explaining the qualities of leaders who regularly practice mindfulness.

As research from the discipline of neuropsychology shows how the benefits of formal meditation as a mindfulness practice can rewire our brain and mind, we believe it is inevitable to integrate mindfulness meditation into our proposed theoretical framework. This has also been acknowledged by other scholars within the discipline of leadership (e.g., Brendel et al., 2016; Kersemaeker et al., 2020; Reitz et al., 2020). We therefore suggest that meditation as a formal practice is the grand foundation of “the three pillars of the mindful leader.”

**Figure 1:**
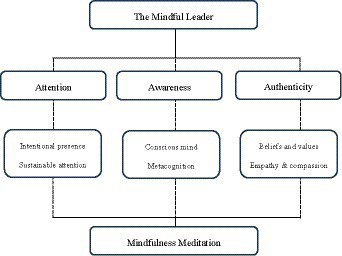
Theoretical framework on the qualities of the mindful leader.

We summarize and model our proposed theoretical framework on the qualities of the mindful leaders in Figure 1. In the following section, we’ll discuss the contributions of our literature review to the leadership discipline on mindfulness.

## 6 Discussions and conclusion

We have striven here to answer the call for a strengthened theoretical foundation of the qualities of the mindful leader as a research phenomenon. Our hope was to reduce the inconsistency and ambiguity on our understanding about the qualities that are likely to develop when leaders practice mindfulness.

Our study is premised on the conviction that any theorizing about a novel phenomenon (Wacker, 1998, Shepherd and Suddaby, 2017) requires a literature review to identify, and analyze, all significant scientific contributions (Webster and Watson, 2002) that have accumulated over time and that offer emerging pieces of evidence and explanations about the phenomenon (Wagner and Berger, 1985), all of which can be synthesized into a convincing theory (Wagner and Berger, 1985) that proposes a framework consisting of the theory’s main concepts and circumstances (Wacker, 1998).

Thus far, previous contemporary studies that have proposed a theoretical framework about mindful leaders have answered the call for greater understanding about qualities that are nurtured by practicing mindfulness. But these studies have documented variations in the qualities developed, and we have therefore been left with an inconsistent and ambiguous understanding about the phenomenon.

These previous studies have also adopted different methodological approaches, many of which often are not grounded in a scientific assumption for building theories, making them appear merely as personal notions of their authors. Some of the studies only offer a conceptualization of mindful leaders’ qualities, without building a theoretical framework that includes main concepts and circumstances (Mohapel, 2018; Donaldson-Feilder et al., 2019). Other studies build a theoretical framework, but does not systematically review and integrate previous studies into their proposed framework (Hunter and Chaskalson, 2013; Ehrlich, 2017; King and Badham, 2018; Mohapel, 2018; Stedham and Skaar, 2019). Contemporary studies therefore do not build a strong theoretical foundation that explains mindful leaders as a phenomenon, which in turn makes it difficult for later studies to be grounded on the extant research literature, to promote further understanding of the phenomenon.

We believe that our own approach more fully answers the call in the literature. At its heart, our review applies a well-established research methodology for theorizing, as we have conducted a well-considered literature review (Webster and Watson, 2002; Wong et al., 2013; Snyder, 2019). Having identified, analyzed, and synthesized the relevant conceptual models, empirical evidences, and theoretical explanations, we have built a theory about the qualities of the mindful leader that we believe expands our scientific knowledge of the novel phenomenon, and that can be effectively implemented in practice (Wacker, 1998; Webster and Watson, 2002; Weber, 2003) as a solution to real contemporary leadership problems that rest on the constant context in chaos (Wacker, 1998; Shepherd and Suddaby, 2017).

When integrating mindfulness meditations as the foundation of our theoretical framework, we implicitly entangle mindfulness as a formal practice that can be applied as a meditation form. Our view differs from some earlier work on the phenomenon, that assume that “mindfulness” is a personality trait of the leader (e.g., Verdorfer, 2016; King and Harr, 2017; Decuypere et al., 2018; Arendt et al., 2019).

Based on the neuroscientific evidence of positive influence of mindfulness mediation on our brain’s default mode network (DMN) and our executive control network (ECF), and on our sympathetic nervous system (SNS) and the parasympathetic nervous system (PNS), and the interoceptive signals flowing from our body to the brain. We are now convinced that, in order to acquire the three main leader qualities, that we associate with the mindful leader, leaders must employ a formal mindfulness meditation practice.

We hope that now that you have read our article, you “*take a deep inbreath, softly let your belly, lungs and chest expand… and on the outbreath, slowly let the breath leave your body through your nose… feel the sensations arising in your body and mind… calm your mind… We invite you to bring this calmness and clarity to your next few moments.*”

## Data availability statement

The original contributions presented in the study are included in the article/supplementary material, further inquiries can be directed to the corresponding author.

## Author contributions

JD: Writing – original draft. HL: Writing – original draft.
